# Analysis of China’s Industrial Green Development Efficiency and Driving Factors: Research Based on MGWR

**DOI:** 10.3390/ijerph18083960

**Published:** 2021-04-09

**Authors:** Ke Liu, Yurong Qiao, Qian Zhou

**Affiliations:** 1 School of Economics and Management & Center for Industry and Innovation, Zhengzhou University of Light Industry, Zhengzhou 450000, China; liuke_liu@163.com (K.L.); qiaoyurong9@163.com (Y.Q.); 2 Economics School, Zhongnan University of Economics and Law, Wuhan 430073, China

**Keywords:** industrial green development, efficiency evaluation, spatial heterogeneity, multiscale geographical weighted regression (MGWR), China

## Abstract

With increasingly severe constraints on resources and the environment, it is the mainstream trend of economic development to reduce industrial pollution emissions and promote green industrial development. In this paper, a super-efficiency slacks-based measure (SBM) model is adopted to measure the industrial green development efficiency (IGDE) of 289 cities in China from 2008 to 2018. Moreover, we analyze their spatiotemporal differentiation pattern. On this basis, the multiscale geographical weighted regression (MGWR) model is used to analyze the scale differences and spatial differences of the driving factors. The results show that the IGDE is still at a low level in China. From 2008 to 2018, the overall polarization of IGDE was relatively serious. The number of high- and low-efficiency cities increased, while that of medium-efficiency cities greatly decreased. Secondly, the IGDE presented an obvious spatial positive correlation. MGWR regression results show that the technological innovation, government regulation, and consumption level belonged to the global scale, and there was almost no spatial heterogeneity. Other driving factors were urbanization, industrial structure, economic development, and population density according to their spatial scale. Lastly, the influence of economic development and technological innovation had a certain circular structure in space; the influence of population size mainly occurred in the cities of the southeast coast and northeast provinces; the influence of urbanization was more obvious in the most northern provinces of the Yangtze River, while that of industrial structure was mainly concentrated in the most southern cities of the Yangtze River Economic Belt (YREB). Spatially, the influence of consumption was manifested as a distribution trend of decreasing from north to south, and the government regulation was manifested as increasing from west to east and then to northeast.

## 1. Introduction

In recent years, global ecological and environmental events have shown a trend of frequent high risks, with economic growth facing the risk of slowing down and growth momentum gradually weakening [1,2]. As a result, the ecological environment and economic situation have become more complicated. In the 13th Five-Year Plan, China gave high priority to ecological protection and proposed to uphold the concept of green development and promote high-quality economic growth. In order to implement the 13th Five-Year Plan and promote industry green development, the state has promulgated the Green Development Plan for Industry (2016–2020) (Ministry of Industry and Information Technology (2016) No. 225), which points out that it is necessary to promote industry green development, improve the level of resource utilization, reduce energy consumption, and vigorously promote industry green development [3]. The year 2020 marks the end of the 13th Five-Year Plan period. According to the government work report, the overall economic performance has been stable, the economic structure has been optimized, the emission of major pollutants has been reduced, the level of green development has been raised, and the ecological environment has been generally improved. However, we should be soberly aware that the current situation facing China’s economy and ecological protection is still very serious. The data showed that the added value of the primary, secondary, and tertiary industries in 2019 accounted for 7.1%, 39.0%, and 53.9% of the gross domestic product (GDP), respectively [4]. Although the industrial structure has been adjusted and optimized, the proportion of secondary production is relatively high, the development mode is still relatively extensive, and the utilization rate is low. Moreover, The National Ecological Environment Quality 2019 Summary showed that (1) the ecological environment quality in China is bad, (2) more than half of the cities perform poorly in ecological quality, and (3) multiple watersheds and lakes are slightly polluted. There are obvious differences in the industrial structure and ecological environment across regions. Therefore, it is necessary to scientifically measure China’s urban green development efficiency and reveal its spatial evolution law. This paper uses the multiscale geographical weighted regression (MGWR) model to measure the differences in scale and space of the driving forces to improve the efficiency of the green development of China’s urban industries. We aimed to test the achievements of the green development of the industry during the 13th Five-Year Plan period and identify the factors affecting industrial green development efficiency (IGDE), thereby guaranteeing smooth sailing of the 14th Five-Year Plan period.

This paper uses a super-efficiency slacks-based measure (SBM) model to measure the IGDE of 289 cities of China and analyzes their spatiotemporal differentiation pattern. On this basis, the MGWR model is used to analyze the scale and spatial differences of the driving factors, so as to provide a reference for improving IGDE and implementing differentiated management strategies in various regions.

### 1.1. Literature Review

Green development originates from ecological economy and green economy [5]. The concept of green development was put forward with the promotion of sustainable development ideas. Sustainable development models such as “ecological economy”, “low-carbon economy”, “circular economy”, and “green economy” all embody the concept of green development from different angles [6,7]. It involves paying attention to environmental protection while ensuring economic and social development; its goals are saving resources and remaining environmental friendly [8]; its critical path is low-carbon industrialization and greening; the development model is to promote the transformation of “industrial civilization” to “ecological civilization”; it emphasizes (1) the symbiosis of economic, social, and natural systems, and (2) system, unity, and coordination. The essential connotation of industrial green development is to respond to “ecological carrying capacity” at the industrial level [9]. It is an industrial development method that focuses on the coordinated development of economic benefits and ecological environmental protection. By constructing an industrial production method with low resource consumption, high economic benefits, low pollution emissions, and high technical content, it can achieve organic coordination among economic development, resources, and the environment [10]. The basis for its determination is to satisfy economic growth while simultaneously reducing ecological damage.

#### 1.1.1. Discussion about Industrial Green Development Efficiency

Efficiency began as a physical concept, mainly used to measure the degree of energy loss. With the continuous crossover and integration of various disciplines in recent years, the term “efficiency” has been widely applied in the study of management, economics, social sciences, and other disciplines, and gradually extended to the ratio of input to output [11]. IGDE is the input–output efficiency of the industry from the green perspective, which reflects the ability to maximize economic output under the dual constraints of low consumption and low emissions. The value of IGDE is the ratio of total input and total output, after considering all factors comprehensively. The selection of indicators is generally divided into three levels: input, desired output, and undesired output [10,12]. Specifically, scholars select indicators on the basis of the characteristics of the research object and considering the continuity and availability of data. Input indicators focus on capital, labor, and energy. Desired output indicators include industrial output, resource recycling levels, and so on. Undesired output mainly focuses on pollutant emissions. Scholars dispute whether environmental indicators should be used as input or output. Some scholars analyzed environmental variables as inputs like capital and labor [13], while others included environmental pollution as a “bad” output in the efficiency evaluation system [14,15]. The research area of IGDE ranges from the macro-national and regional level to the micro-industrial park level [16,17,18], as well as from the overall industry to specific industries, such as the paper industry, power industry, and agriculture [19,20]. The research methods of IGDE mainly include data envelopment analysis (DEA) and stochastic frontier analysis (SFA). DEA method is a nonparametric method which is more widely adopted because it does not depend on the establishment of a growth function and the model has abundant variation forms [21,22,23].

#### 1.1.2. Influencing Factors of Industrial Green Development Efficiency

At present, there are abundant research achievements related to IGDE, including ecological efficiency [24], green innovation efficiency [25], and IGDE [26]. It has been pointed out that industrial structure, human capital, opening up, environmental control, and urbanization have significant effects on IGDE. Among them, city size and industrial agglomeration show a U-shaped relationship with green efficiency. Technological innovation, urbanization foreign direct investment, and economic development are conducive to improving IGDE, among which the role of economic development is extremely outstanding [27]. However, human capital and degree of openness show a negative impact [28]. Some studies stated that there is no significant relationship between environmental regulation and industrial ecological efficiency [29], but others showed that the interaction between government intervention and environmental regulation has a significant impact on green development [30]. Although the industrial structure has a negative impact, there is no denying the positive promotion effect of the rationalization of industrial structure and upgrading of industrial structure [28]. Therefore, scholars mostly propose ways to improve IGDE from the aspects of adjusting industrial structure, rational industrial layout, and increasing technological innovation [31].

#### 1.1.3. Summary

To sum up, rich research results have been obtained. They provide references for this paper in terms of green development efficiency and related studies. However, there are still some aspects to be improved. Firstly, there are many studies on ecological efficiency, green innovation efficiency, and IGDE, but few in-depth discussions on IGDE. Secondly, existing studies provide a good reference for the study on green development efficiency, but relevant indicators still need to be optimized in combination with research purposes. Thirdly, although the literature has carried out a quantitative analysis of the IGDE, its efficiency and the influence factor’s spatiotemporal measure aspect are still relatively deficient.

In this paper, we objectively evaluate IGDE and analyze the scale differences and spatial differences of its driving factors by using the MGWR model in 289 cities of China. The main aims were to (1) scientifically measure IGDE and analyze the spatial characteristics using the spatial autocorrelation theory, and (2) explore the spatial differences of the influencing factors of IGDE and provide data support for the research and practice of green industry.

## 2. Materials and Methods

### 2.1. Research Methods

#### 2.1.1. Super-Efficiency SBM

DEA is widely used to measure relative efficiency between multiple inputs and outputs. It is more applicable to the research of complex economic systems without presetting a specific functional form. However, the traditional DEA method is a radial and angle model. In the process of measuring IGDE, slack variables are usually not taken into account. The radial model will overestimate efficiency, while the angle model will cause result deviation due to the neglect of some indices. In addition, the traditional DEA ignores the non-expected output index, which violates the practical significance of efficiency evaluation and leads to biased measurement results. The slacks-based measure (SBM) model proposed by Tone effectively solves the defect of traditional DEA, but it cannot realize the further comparison of multiple effective units. Therefore, combining the advantages of both, Tone [32] proposed the super-efficiency SBM model, which perfectly solved the above problems. In the process of industrial development, the input of capital and manpower produces economic benefits and inevitably produces byproducts (wastewater, waste gas, waste residue, etc.). Therefore, to scientifically and reasonably calculate IGDE, this paper adopts the super-efficiency SBM model containing the undesired output. The model is constructed as follows:

(1) $$\begin{array}{l} Min\rho =\frac{\frac{1}{m}\sum_{i=1}^{m}\left(\bar{x}/x_{ik}\right)}{\frac{1}{r_{1}+r_{2}}\left(\sum_{s=1}^{r_{1}}\bar{y^{d}}/y_{sk}^{d}+\sum_{q=1}^{r_{2}}\bar{y^{u}}/y_{qk}^{d}\right)}\\ s.t.\left\{ \begin{array}{l} \bar{x}\geq \sum_{j=1,\neq k}^{n}x_{ij}\lambda_{j};\bar{y^{d}}\leq \sum_{j=1,\neq k}^{n}y_{sj}^{d}\lambda_{j};y^{d}\geq \sum_{j=1,\neq k}^{n}y_{qj}^{d}\lambda_{j};\\ \bar{x}\geq x_{k};\bar{y^{d}}\leq y_{k}^{d};\bar{y^{u}}\geq y_{k}^{u};\\ \lambda \geq 0,i=1,2,\cdots ,m;j=1,2,\cdots ,n;j\neq 0\\ s=1,2,\cdots ,r_{1};j=1,2,\cdots ,r_{2}; \end{array}\right. \end{array}$$

where it is assumed that there are *n* decision-making units (DMUs), and each DMU consists of input *m*, expected output *r*_1_, and unexpected output *r*_2_. *x*, *y^d^*, and *y^u^* are the elements in the corresponding input matrix, expected output matrix, and unexpected output matrix, while ρ is the value of IGDE.

#### 2.1.2. Spatial Cold/Hot Spot Analysis

Exploratory Spatial Data Analysis (ESDA) is usually used to study the distribution characteristics of spatial data and reflect the spatial dependence or heterogeneity of geographical phenomena [33]. This paper selected the global Moran’s I index to understand the overall spatial characteristics of IGDE and analyzed its spatial agglomeration situation. The Getis–Ord G_i_* index reflects the local spatial correlation and difference degree of IGDE. Its calculation formula is as follows:

(2) $$I=\frac{\sum_{i=1}^{n}\sum_{i=1}^{m}w_{ij}\left(x_{i}-\bar{x}\right)\left(x_{j}-\bar{x}\right)}{S^{2}\sum_{i=1}^{n}\sum_{i=1}^{m}w_{ij}}.$$

(3) $$G_{i}^{*}=\frac{\sum_{j=1}^{n}w_{ij}(d)x_{j}}{\sum_{j=1}^{n}x_{j}}.$$

In Equation (2), *I* is the global Moran’s I, *n* is the number of regions, *x_i_* and *x_j_* represent efficiency values in region *i* and region *j*, respectively, and *W_ij_* is the spatial weight matrix.

In Equation (3), the Z test for G_i_* shows that Z(G_i_*) is positive and the value is significant, indicating that the values around position *i* are greater than the mean and belong to the high-value spatial clustering (hot spot). Otherwise, they belong to the low-value spatial clustering (cold spot).

#### 2.1.3. Multiscale Geographically Weighted Regression (MGWR)

The specific bandwidth of each variable in the MGWR model can be used as an indicator of the spatial scale of the action of each spatial process, which is closer to a real and useful spatial process model [34]. The calculation formula of the MGWR model is as follows:

(4) $$y_{i}=\overset{k}{\underset{j=1}{\sum }}\beta_{bwj}\left(u_{i},\;v_{i}\right)x_{ij}+\varepsilon_{i},$$

where *y_i_* is the global dependent variable, *x_ij_* is the independent variable, *bwj* represents the bandwidth used by the regression coefficient of the *j*-th variable, (*u_i_*, *v_i_*) is the spatial coordinate of the *i*-th region, and *ε_i_* is the random error term. Each regression coefficient *β_bwj_* is based on local regression, and the bandwidth is specific. This is the biggest difference between MGWR and classical Geographically Weighted Regression (GWR).

### 2.2. Variable Selection

#### 2.2.1. Calculation of Industrial Green Development Efficiency

Table 1 shows the selection of indicators for IGDE. On the basis of the relevant studies on the current input–output IGDE, this paper selects labor and capital as input variables [35], the GDP of the three industries as expected output, and the main environmental pollution source as unexpected output. Specifically, there are many measures of capital in the existing research, such as fixed asset investment and net value of fixed assets. This paper chooses the data of total fixed asset investment of the whole urban society as the capital input index. The labor force is the basis of the green development of the industry. When measuring this index, we use the sum of two indicators: number of urban employees at the end of the year and number of urban private and individual employees. The expected output is usually the regional GDP. The discharge of industrial wastewater, discharge of industrial SO_2_, and discharge of industrial smoke and dust were selected as data to measure the main pollutants in industrial production. We calculated unexpected output through the discharge of industrial wastewater, discharge of industrial SO_2_, and discharge of industrial smoke and dust using the entropy method.

According to the above measurement model of IGDE, this paper took 289 cities as samples and selected relevant data of these urban input and output indicators to calculate IGDE. When selecting relevant indicators, the operability of data should be considered. Secondly, the metric nature of the indicators should be considered to ensure comparison and analysis. Therefore, the index data of this paper were mainly from The China City Statistical Yearbook from 2009 to 2019, the statistical yearbook of provinces, and the data center of the Ministry of Environmental Protection.

#### 2.2.2. Influencing Factors

The IGDE is an index to evaluate the industrial development efficiency of a country or region considering the resource input and environmental cost. Referring to the relevant research, the factors affecting IGDE mainly include the following:

(1) Economic development. Economic development is measured by per capita GDP, which has the effect of funding support for technological innovation. Rapid economic development can provide more funds for improving industrial green technology, optimizing energy structure, and investing in environmental governance, so as to promote IGDE [36].

(2) Consumption level. Residents with a higher consumption level are more aware of environmental protection, which can stimulate the demand for green products and encourage enterprises to improve the traditional production technology and manufacturing process [37]. This paper adopts the amount of per capita social consumer goods to represent the consumption level.

(3) Technological innovation. Technological innovation is conducive to improving production technology. While improving production efficiency, it can reduce pollution emissions in industrial production and enhance the driving force of industrial green development [38]. Therefore, the proportion of science and education investment in general public expenditure is adopted to measure the level of technological innovation.

(4) Industrial structure. The secondary industry is the industrial sector which consumes the most resources and damages the ecological environment the most seriously. The process of industrialization is inevitably accompanied by the massive consumption of resources and the serious pollution of the ecological environment [39]. Therefore, the proportion of the secondary industry is selected to measure industrial structure.

(5) Population density. On one hand, the concentration of population may provide sufficient labor force for industry development; on the other hand, it has an inverted U-shaped effect on environmental pollution [40]. Therefore, the ratio of total population to administrative area is used to measure population density.

(6) Urbanization level. The improvement of urbanization level promotes the interrelation between industries, promotes the improvement of industry-related infrastructure, helps to gather high-end production factors and the labor force, and promotes the high-quality development of industries [41]. This paper measures the indicator by the urbanization rate.

(7) Government regulation. The government plays a guiding and restraining role in the green development of industry. By guiding industrial transformation and upgrading and regional industrial division, the supervision of pollution prevention and control, energy conservation, and emission reduction can be strengthened, so as to improve IGDE [42]. Therefore, the ratio of fiscal expenditure to regional GDP is used to measure the intensity of government regulation.

(8) Openness. According to Wang et al. [43], the ratio of actual utilized foreign capital and GDP represents the degree of openness. On one hand, the introduction of foreign capital may introduce more pollution-intensive industries with backward foreign production capacity [44]; on the other hand, a higher openness degree leads to a more obvious spillover effect of technology. This can improve the green production efficiency of the industry by the way of green production capacity and environmental governance capacity.

## 3. Results

### 3.1. Spatiotemporal Pattern and Evolution Analysis of Industrial Green Development Efficiency

On the basis of the input–output data of 289 cities of China, this study uses the super-efficiency SBM model with undesired output to measure IGDE. In order to fully understand its change and spatial distribution, we analyze it from the perspectives of region, province, and city.

#### 3.1.1. Analysis of Regions

**Figure 1:**
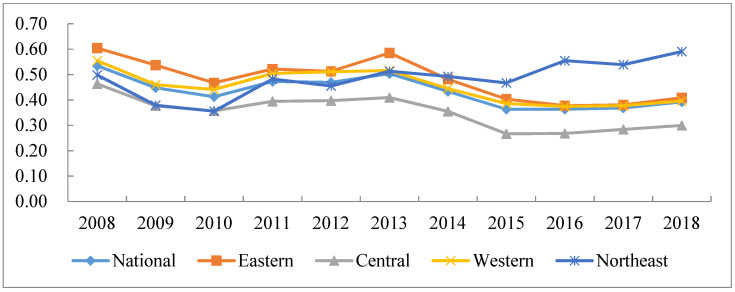
Changes in IGDE in China from 2008 to 2018.

As presented in Figure 1 and Table 2, green development still presents low efficiency in China. Only one point was greater than 0.6. Therefore, there are substantial efforts to improve IGDE in China. In terms of fluctuation, all regions show different degrees of decline except for the northeast. The eastern and the central regions show the most obvious drops, reaching 32.44% and 35.41%, respectively. Specifically, the northeast region showed a “decline followed by a rise in fluctuation” with an obvious increase, dropping to the region with the lowest efficiency value from 2008 to 2010, and then continuously rising to the region with the highest efficiency value from 2011 to 2018. Moreover, the area where efficiency value drops was mainly manifested as an initial drop before steadily rising, with a relatively fast decline rate. According to the regional efficiency value, without considering the northeast, the IGDE ranking was as follows: eastern > western > national > central; then, considering the efficiency value of the northeast area, the efficiency value in the region in 2008–2010 was slightly lower than the national level. From 2011 to 2013, it kept rising and exceeded the national level, but was slightly lower than the western region. After 2014, the efficiency value surpassed the western and eastern regions and become the region with the highest efficiency value.

#### 3.1.2. Analysis of Provinces

**Figure 2:**
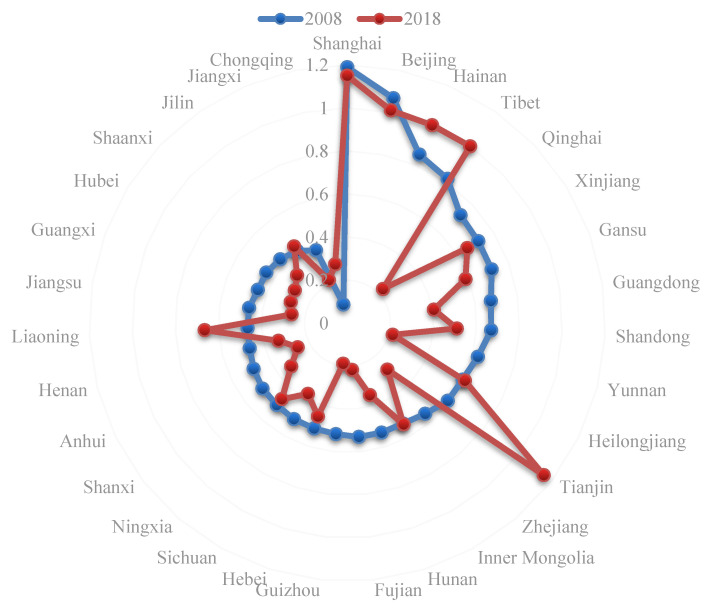
Comparison chart of provincial industries green development efficiency in 2008 and 2018.

According to Figure 2, the IGDE increased in some provinces (Tianjin, Liaoning, Chongqing, Tibet Autonomous Region, Hainan, Jilin, Heilongjiang, and Inner Mongolia Autonomous Region) in 2018 compared with 2008, while it decreased to different degrees in other provinces, presenting a “contraction” trend on a whole, consistent with regional changes. In 2008 and 2018, the efficiency values of Beijing and Shanghai were always greater than 1, ranking top three and above the production frontier. Provinces with efficiencies below 0.5 were mostly in the central and western regions (Jilin, Henan, Shanxi, Shaanxi, Hubei, Guangxi, Chongqing, Jiangsu, Anhui, and Jiangxi). Among them, Chongqing in 2008 and Jiangxi in 2018 had the worst efficiency, with values of 0.0876 and 0.2167, respectively. There was a serious problem of redundancy in resource investment. In addition, there was a large range of changes in Tianjin and Qinghai. The efficiency value of Tianjin increased from 0.5910 to 1.1552, and that of Qinghai decreased from 0.7266 to 0.2289.

#### 3.1.3. Analysis of Cities

**Figure 3:**
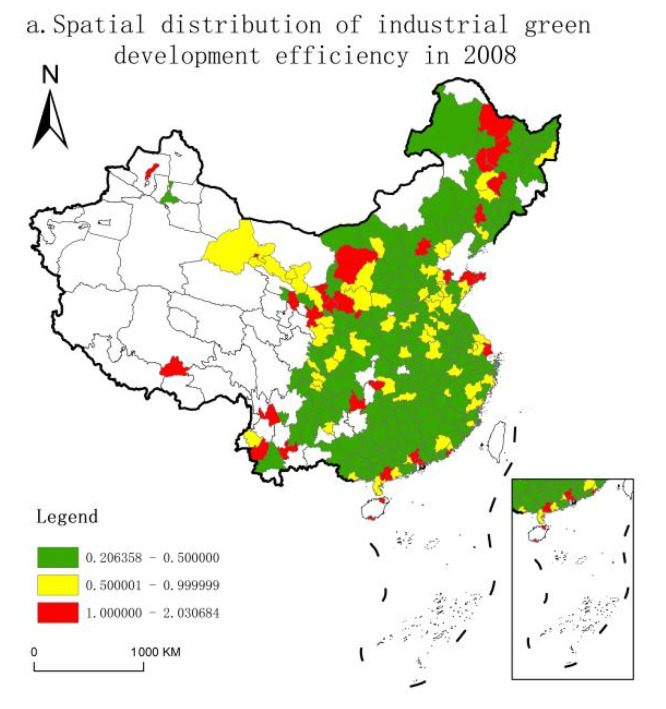

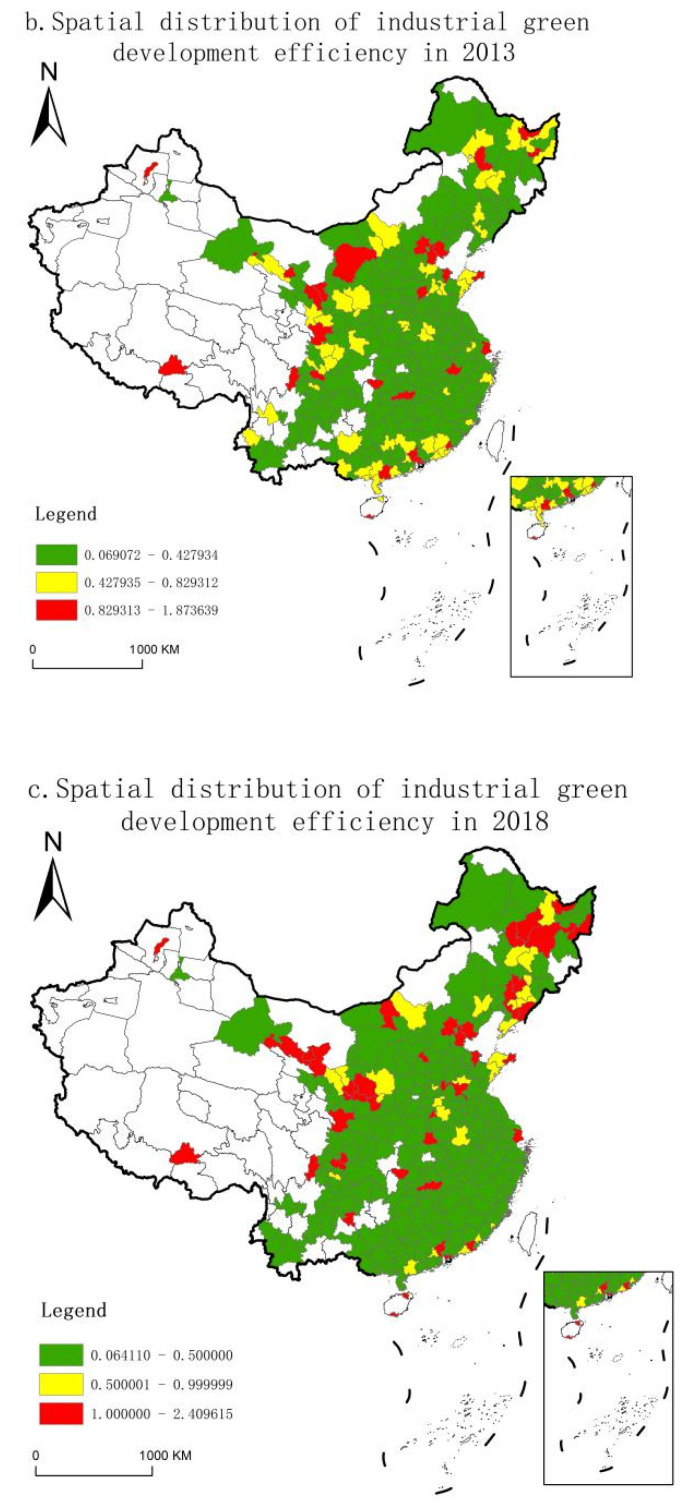
Spatial distribution of IGDE in 2008, 2013, and 2018.

We selected the efficiency values of 2008, 2013, and 2018 to observe the relative change trend of IGDE of cities. We divided the sample into three ranges: high efficiency (ρ ≥ 1), medium efficiency (0.5 < ρ < 1,) and low efficiency (ρ ≤ 0.5). In addition, ArcGIS10.3 was used to visually express the efficiency value, and the distribution map of IGDE of China’s cities was obtained (Figure 3). First, from the interval divided by the efficiency value, the number of cities in the high- and low-efficiency ranges increased, while the number of medium-efficiency cities greatly decreased, while the polarization was relatively serious on a whole. Specifically, the number of high-efficiency cities increased from 20 in 2008 to 27 in 2018, cities in the low-efficiency range increased from 163 to 233, and those in the medium-efficiency range decreased by 72.64%. Second, from the perspective of regional changes, in 2008, cities with high and medium IGDE were mainly distributed in northeast China, western China, and eastern coastal regions, while cities with low efficiency were mainly concentrated in Shanxi, Henan, Hubei, Hunan, Jiangxi, and other central provinces. By 2013, the IGDE in Fujian, Zhejiang, Gansu, and Hunan decreased, the efficiency of Heilongjiang improved, and some cities with high efficiency in the Yangtze River Economic Belt (YREB) and the Beijing–Tianjin–Hebei region emerged. By 2018, cities with an IGDE of more than 1 were mainly concentrated in northeast and northwest China, the Beijing–Tianjin–Hebei region, and the YREB. The most obvious change was the surge in low-efficiency cities, accounting for 80.62% of the whole country, widely distributed in various regions.

### 3.2. Spatial Exploration of Industrial Green Development Efficiency

**Figure 4:**
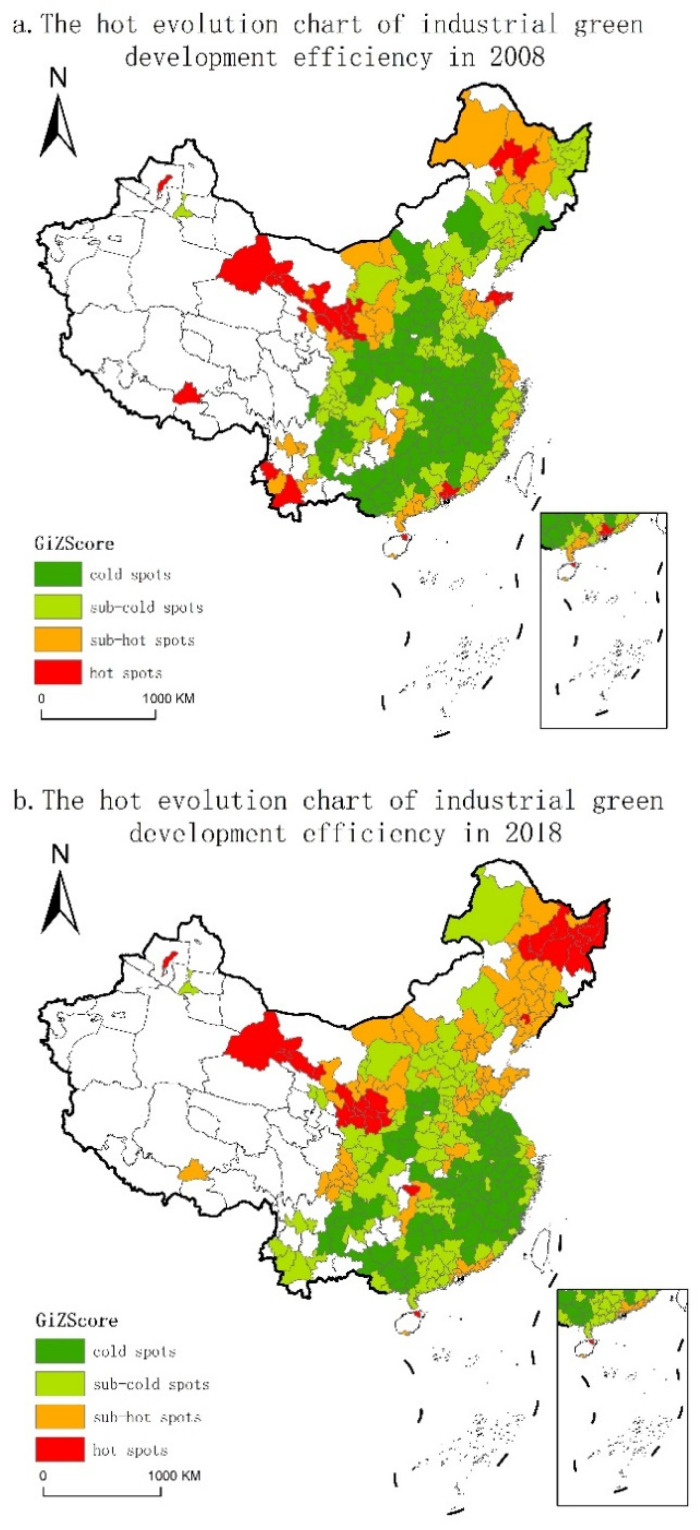
Cold/hot spot evolution of IGDE.

This paper further analyzes the spatiotemporal evolution characteristics of IGDE in China through ESDA. Global spatial autocorrelation was used to test the overall trend of spatial correlation of adjacent cities in the whole research area, which was mainly measured by Global Moran’s I index. The indices from 2008 to 2018 were calculated as 0.0363, 0.0390, 0.02476, 0.0404, 0.0302, 0.0523, 0.0408, 0.0560, 0.0763, 0.0785, and 0.1027, respectively, all of which passed the test at the level of 99% confidence, indicating that IGDE has a significant positive spatial autocorrelation. At the same time, in order to make up for the deficiency of global autocorrelation and better reflect the clustering degree of high and low values in local areas, the Getis–Ord G_i_* index was used for hot spot analysis. Through the natural fracture method, we divided the hot and cold spots into four categories (hot area, sub-hot area, sub-cold area, and cold area) and generated the spatial evolution figures (Figure 4).

From the evolution figures of spatial hot/cold spots of IGDE, in 2008, hot spots were mainly distributed in some cities in the western, northeast, and eastern regions, accounting for 8% of the total number. To be specific, the hot spots in the west included Gansu, Ningxia, and the southwest of Yunnan, while the eastern areas were concentrated in Guangdong and Shandong. By 2018, the number of hot spots increased in northeast China and the spatial distribution changed, being mostly concentrated in the northern region. In addition, the proportion of sub-hot cities increased from 17.30% to 28.03%. From the perspective of cold spot regions, there were 108 cities in 2008, which were widely distributed in Shanxi, Hubei, Hunan, Anhui, Jiangxi, Jiangsu, and Guangxi. By 2018, the number of cold spots dropped to 81 cities, showing a shrinking trend. To be specific, most cities in Shanxi and Henan changed from cold spots to sub-cold spots, and most cities in Liaoning and Jilin changed from cold spots or sub-cold spots to sub-hot spots. On the whole, during the research period, the hot and cold zones in the IGDE had a significant spatial concentration, and there were obvious temporal and spatial changes. The hot spots showed a trend of expansion, while the cold spots showed a trend of contraction.

### 3.3. Spatial Pattern Analysis of Driving Factors

#### 3.3.1. Model Comparison

In this paper, the IGDE was taken as the dependent variable, and economic development, consumption level, technological innovation, industrial structure, population density, urbanization level, government regulation, and openness level were taken as the independent variables. MGWR 2.2 software was used to calculate the regression coefficients of each driving factor and analyze its spatial heterogeneity, and the results are presented in Table 3. Compared with Geographically Weighted Regression (GWR) and Ordinary Least Squares (OLS), MGWR has a higher goodness-of-fit *R*^2^ and a lower Akaike Information Criterion (AICc) value, showing its superiority to the other two models. In terms of residual sum of squares and effective parameters, MGWR performs better and uses fewer parameters to get a regression result closer to the true value. Therefore, the MGWR model was selected in this study to explore the spatial heterogeneity of the factors affecting IGDE.

#### 3.3.2. Scale Analysis

Compared with the fixed scale of classical GWR, the main advantage of MGWR is the differential scale of different variables, i.e., the bandwidth of each variable is specific. According to Table 4, the GWR bandwidth was 140, accounting for 48.44% of the total sample. In contrast, MGWR results showed that the bandwidth of different variables varied greatly. In MGWR regression results, all regression coefficients were significant except for openness. Specifically, the MGWR bandwidths of technological innovation, government regulation, and consumption level were 288, 276, and 287, respectively, which belong to the global scale, and there was almost no spatial heterogeneity. In other words, the IGDE in all regions was basically the same under the influence of technological innovation, government regulation, and consumption level. The scales of urbanization level and industrial structure were 167 and 131, respectively, accounting for about 50% of the total sample volume. The coefficient varied in space, while it was relatively stable on a whole. The scales of economic development and population density were relatively small (92 and 82, respectively), which were closer to that of urban agglomeration. There was a large difference in space, indicating that the coefficients were basically the same on this scale; however, drastic changes would occur if they exceeded this scale.

#### 3.3.3. Spatial Pattern Analysis of Coefficients

MGWR was used for regression, and the statistical description of each regression coefficient is shown in Table 5. The influence of each variable on different urban IGDE presented a specific regression coefficient, with big differences. This indicates that the influence of each variable in different cities varied greatly. The mean value reflects the average level of the contribution degree of influencing factors to IGDE. From the positive and negative aspects of the mean coefficient, the impact of economic development, government regulation, population density, and consumption level on the IGDE was positive, while other variables were negative. In terms of the absolute value of the mean coefficient, economic development had the strongest influence, followed by government regulation and consumption level, and openness had the weakest influence.

**Figure 5:**
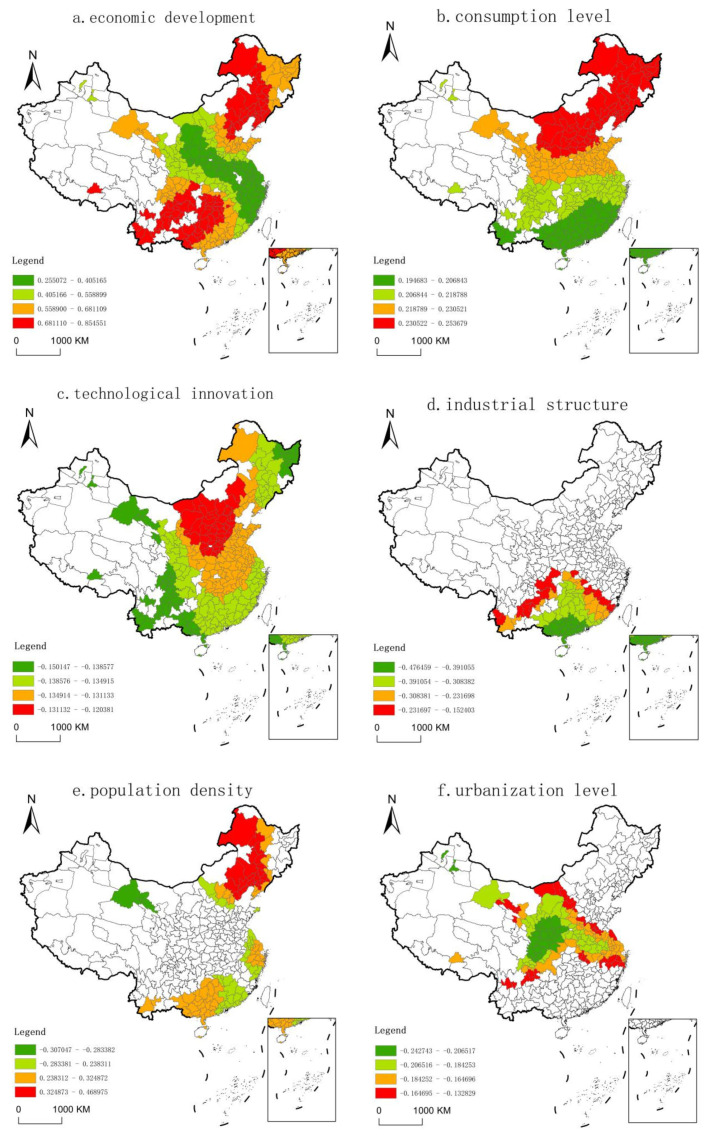

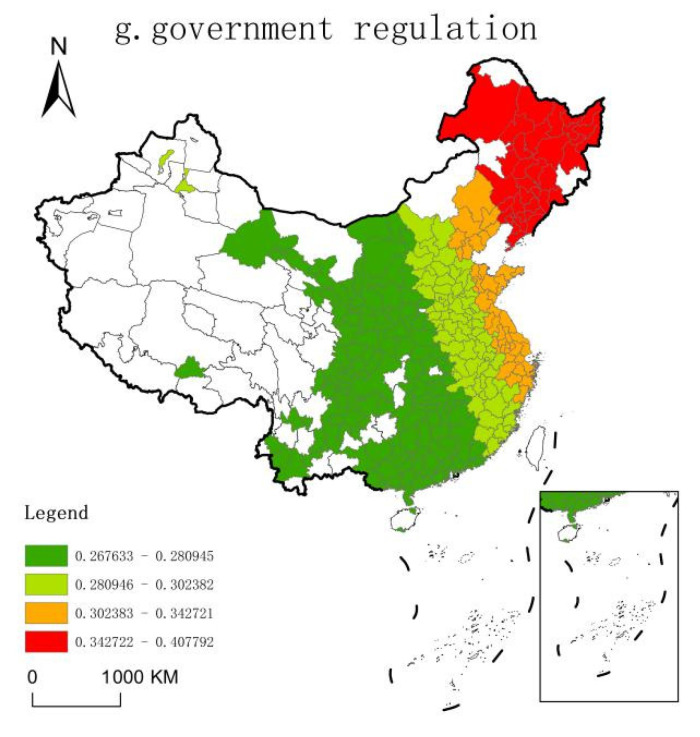
Spatial distribution of influencing factors.

Economic development (Figure 5a). Economic development had a significant positive impact on IGDE. This is because, with the increase in economic development, the public’s awareness of environmental protection increases, and the increase in income provides support for the public to buy relatively highly priced green products. In the meantime, enterprises pay more attention to social responsibility and tend to adopt environment-friendly technologies and develop green products to meet consumer demand. The regression coefficient was between 0.2526 and 0.8545. Its influence on space had a certain ring-layer structure. The areas with higher regression coefficients were concentrated in the northeast economic circle, Bohai economic circle, Pearl River Delta economic circle, Beibu Gulf economic circle, Chengdu–Chongqing economic circle, and southwest economic circle. The low-value areas were concentrated in the Yangtze River Delta economic circle, Haixi economic circle, and part of the central emerging economic circle. The reasons for this distribution may be as follows: first, for high-value areas, the current new round of the northeast revitalization strategy initially reversed the situation of continuous economic decline, and industrial transformation and upgrading promoted high-quality economic development, which is conducive to promoting IGDE. Second, for the low-value areas, the Yangtze River Delta economic circle is geographically superior, and the IGDE is relatively high or has been in the production frontier. The efficiency improvement brought by economic development is limited. The emerging economic circle in central China had a strong development momentum in recent years. However, the problem that cannot be ignored is that the industrial level is low, and the development mode is relatively extensive, which affects the economic quality and efficiency. Therefore, the improvement of economic development cannot significantly promote IGDE.

Consumption level (Figure 5b). The regression coefficient of consumption level for the IGDE was between 0.1949–0.2537, and the influence degree showed a distribution trend of gradual weakening from north to south. To be specific, compared with the cities in the YRBE and those in the south, the improvement of consumption level in the cities in the north of the Yangtze River basin significantly improved IGDE. This is closely related to the following factors: first, production and consumption promote each other, and green production and green consumption are closely linked. With the improvement of consumption level, consumers have a generally stronger awareness of green consumption. In order to cater to consumption, enterprises should strengthen green technology, improve innovation ability, and further improve the IGDE. Second, compared with the south, the consumption in the north is relatively low, and the improvement of consumption has a greater marginal impact on the improvement of IGDE, thus forming a distribution pattern with a high level in the south and low level in the north.

Technological innovation (Figure 5c). The regression coefficient of technology innovation for IGDE was between −0.1501 and −0.1217. Since it is a global variable, the overall spatial variation of its influence was small. However, there was a slight difference, which was manifested as the increasing pattern of U-shaped circles with Shanxi, northern Henan, Hebei, and northern Shaanxi as the core. The areas with relatively strong negative influence were mostly distributed in the western regions such as Xinjiang, Sichuan, Yunnan, Gansu and Guizhou, and the east of Heilongjiang. Among them, technology innovation in western cities such as Karamay, Urumqi, Lhasa, Jiuquan, and Lincang had the greatest reverse effect on the IGDE, while the negative influence of cities in north, central, and east China was relatively weak. This is contrary to our theory that increased investment in science and technology will promote innovation and industrial upgrading. We believe that, first, due to the differences in industrial foundation, industrial structure, and technological development among different regions, technological investment has different influences on the IGDE. Second, the input of science and technology in a region is one of the important indices of the innovation index. However, due to the complexity of the system of science and technology innovation, the input–output process has a certain time lag and a long-term effect. Therefore, the input of science and technology in that year may not improve IGDE in time. Third, although the proportion of science and technology input in GDP of all regions has increased in recent years, the transformation of China’s scientific and technological achievements is still in the initial stage of market-oriented reform, and the social innovation environment cannot provide effective support for basic research and transformation of achievements. As a result, the process of scientific and technological input–output is not smooth, the conversion rate is low, and the IGDE cannot be effectively improved.

Industrial structure (Figure 5d). The influence of industrial structure is mainly concentrated in most cities (79 cities) in the south of the YREB, while the influence of the rest of the region does not exist. The regression coefficient is between −0.1670 and 0.0275. Specifically, the industrial structure of 23 cities, including Pearl River Delta and the southeast of Guangxi, had a positive impact on IGDE. This is because Pearl River Delta region was the earliest region to carry out reform and opening up, with a relatively developed manufacturing industry. Its industrial development experienced the stage of accepting the transfer of Hong Kong and international processing and manufacturing industries, as well as the growth stage of localized industries oriented by domestic demand, and it is now moving into the stage of advanced manufacturing with independent innovation. Advanced manufacturing has high added value but less pollution. Therefore, the increase in the proportion of secondary production will positively affect IGDE. The remaining 56 cities had a negative impact mainly because, on the one hand, the large total industrial energy consumption and pollutant emission, as well as a higher proportion of urban secondary industry, led to greater pollution emissions and a more direct negative impact on the industrial green development. On the other hand, the unreasonable industrial structure and backward green production technology led to a low resource utilization rate and low level of clean production, and it failed to give full play to the supporting role of technology in IGDE.

Population density (Figure 5e). The influence of population density was mainly concentrated in 116 cities, including Gansu, northeast China, Beijing–Tianjin–Hebei, the pan-Yangtze River Delta, and the pan-Pearl River Delta, with coefficients of −0.3070–0.4690. The remaining cities basically had no influence. Specifically, only three cities (Jiuquan, Jiayuguan, and Zhangye) had a negative impact on IGDE. This may be because the population carrying capacity is low. The pan-Yangtze River Delta and the pan-Pearl River Delta are two powerful engines of economic development, with superior economic geographical location and strong population pull force for the whole country. Therefore, the population density of these cities has a significant impact on the IGDE. Increasing the population density of northeast China will greatly improve the IGDE. This may be because the economic development of northeast China is highly dependent on the heavy metal industry and resource industry, and simplification of the industrial structure would restrict the employment choice of talents, resulting in serious population loss.

Urbanization level (Figure 5f). The impact of urbanization level on IGDE was obvious in most provinces to the north of the Yangtze River, with coefficients of −0.2427–0.0275. There was no impact on northeast China and southern China. As for the effect of urbanization on IGDE, the jury is still out. On the one hand, with the improvement of urbanization level, population and industries began to form economic agglomeration in cities. Upon improving labor production efficiency and economic output, environmental quality deterioration is accelerated and industrial green development becomes inefficient. On the other hand, with the increase in pollution, the public awareness of environmental protection begins to awaken, public opinion begins to focus on environmental pollution, stricter environmental regulation measures begin to be implemented, the pace of industrial structure transformation and upgrading is accelerated, and more attention is paid to industrial green development.

Government regulation (Figure 5g). The regression coefficient of government regulation ranged from 0.2676 to 0.4078. This variable is a global variable, and its influence degree changed little in the whole space, manifested as the distribution trend of increasing from west to east and then to northeast. The most influential cities were mainly in northeast China, followed by Beijing–Tianjin–Hebei, Shandong Peninsula, and the pan-Yangtze River Delta, and the cities with relatively weak influence were in the central and western regions. The reasons for this distribution may be as follows: the strategies of northeast rejuvenation, the Beijing–Tianjin–Hebei region, and Yangtze River Delta integration have provided strong policy support for these regions. In particular, the revitalization strategy has reversed the trend of economic decline in northeast China and increased expected output, thereby improving the IGDE.

## 4. Discussion

This paper evaluated the IGDE of 289 cities in China from 2008 to 2018, analyzed their spatial and temporal differentiation patterns, and introduced the MGWR model to deeply investigate the scale differences and spatial differentiation of driving factors.

### 4.1. Contribution

First of all, IGDE in China is at a low level, and there are large differences between different regions. The ranking of efficiency is as follows: eastern > western > national > central. This is consistent with existing research [45]. In addition to analyzing the IGDE of regions and provinces, this article put more emphasis on analyzing the efficiency value and spatial distribution of cities from the perspective of prefecture-level cities and found that the two-level differentiation of IGDE is more serious.

Secondly, as for the research on the influencing factors of IGDE, some studies focused on the role of space [46], but few studies adopted the MGWR model to explore the degree and direction of influence of influencing factors on different units. Through research, this paper found that the scale of impact of different variables on the IGDE is quite different. The levels of technological innovation, government regulation, and consumption belonged to the global scale, and there was almost no spatial heterogeneity. The scale of urbanization and industrial structure was half of the sample, and the regression coefficients varied in space, but the overall scale was relatively stable. The spatial impact of economic development and population density was closer to that of urban agglomerations.

Lastly, the positive effects of economic development, consumption levels, population density, and government regulation, and the negative effects of urbanization and industrial structure were all confirmed in existing studies [12,47,48,49]. Studies also showed that technological innovation will promote IGDE [50]. However, this article did not find the promotion of technological innovation, which is consistent with other research results [51]. Studies proved that openness promotes IGDE [50]; however, in this study, it was found that the effect of openness is not significant.

### 4.2. Limitations

The research still has certain limitations and should be further deepened. First, when constructing the IGDE indicator system, this article did not consider all undesired outputs, such as greenhouse gases, resource consumption, and solid waste, because it is difficult to obtain data for most prefecture-level cities in China. Considering the integrity and continuity of the data, we have to discard these data. If there are suitable and available indicators in the future, the existing indicators can be improved. Second, this paper mainly studied the green development efficiency of China’s industries and did not subdivide different industries, such as the service and agriculture industries. In the future, we can conduct research on the green development efficiency of different industries in order to improve their green development efficiency. Third, in view of the limitations of research methods, this paper selected cross-sectional data to analyze the spatial heterogeneity of the IGDE and influencing factors in China; however, it lacks an analysis of the dynamic evolution of time and space. In the future, appropriate research methods will be selected to conduct a more in-depth spatiotemporal dynamic analysis of the IGDE factors.

## 5. Conclusions

This paper calculated the IGDE of 289 cities in China from 2008 to 2018, analyzed their spatial and temporal differentiation patterns, and introduced the MGWR model to deeply investigate the scale differences and spatial differentiation of driving factors. The research conclusions are as follows:

(1) From the regional perspective, IGDE is at a low level in China. All regions showed different degrees of decline except for the northeast region, while the east and the central regions showed the most obvious decline. The ranking of IGDE was as follows: northeast > eastern > western > national > central region. From the perspective of provinces, the IGDE in 2018 increased in some provinces and decreased in others compared with 2008, presenting a “contraction” trend. This was consistent with regional changes. From the perspective of cities, from 2008 to 2018, the number of high-efficiency and low-efficiency cities increased, while that of medium-efficiency cities greatly decreased. There was a serious polarization.
(2) The IGDE presented obvious positive spatial correlation. Compared with OLS and GWR, the MGWR model can better analyze the spatial heterogeneity of the influencing factors. In the MGWR regression results, all variables were significant except for openness. Technological innovation, government regulation, and consumption level belonged to the global scale, with almost no heterogeneity in space. Other influencing factors with spatial heterogeneity were urbanization, industrial structure, economic development, and population density.
(3) The influences of economic development, government regulation, population density, and consumption level on IGDE were positive, while other variables were negative. In terms of the absolute value of the mean coefficient, economic development had the strongest influence, followed by government regulation and consumption level, and openness had the weakest influence. The spatial influence of economic development and technological innovation had a certain circle structure. The influence of population density was mainly concentrated in Gansu, northeast China, the Beijing–Tianjin–Hebei region, the pan-Yangtze River Delta, and the pan-Pearl River Delta. The impact of urbanization level is obvious in most provinces north of the Yangtze River, while the impact of industrial structure is mainly concentrated in most cities south of the YREB. The influence of consumption level was manifested as a distribution trend of decreasing from north to south, and the government regulation had a trend of increasing from west to east and then to northeast.

### Policy Suggestions

The economic development mode should be transformed and residents’ green consumption should be promoted. The purpose of economic growth is to achieve full employment and improve people’s consumption level. Expanding consumption can accelerate economic re-growth and industrial transformation and upgrading, so as to realize a virtuous circle of economic development. Therefore, promoting the high-quality development of green economy and actively cultivating green consumption are effective ways to realize green development. On the one hand, high-quality economic development should be promoted, guided by green development. Green development should be placed as the core, the energy efficiency of resource utilization, pollution control, environmental quality, and ecological construction should be improved, the establishment of a green system the construction of industries, and the production mode of high technology should be accelerated, low resources consumption, little environmental pollution, and IGDE should be promoted, and the health and sustainable development of the economy should be achieved. On the other hand, the concept of green development, green production, and green consumption should be established, and the development of green consumption should be promoted. First, laws and regulations promoting green consumption should be improved and social norms advocating green consumption should be created; supervision of the green consumption market should be strengthened to severely crack down on “pseudo-green” products and other illegal behaviors in the market. Second, the production and sale of green products should be encouraged by reducing or exempting taxes, subsidies, and government procurement, the support of technological innovation for green development should be increased, and consumers should be guided to buy green products. Third, green culture should be actively cultivated, the public’s ecological awareness of resource conservation and environmental protection should be raised, and the whole society should be guided to form a habit of green living and green consumption. The economic development between cities is uneven, and the level of consumption varies greatly between the north and the south. This leads to spatial differences in the impact of economic development and consumption on the IGDE. For cities in the northeast region, although the IGDE is relatively high, the economic development is relatively backward, the pressure of transformation is high, and the ecology is relatively fragile. In the future, when improving economic development, more attention should be paid to the protection of the ecological environment in order to greatly enhance the IGDE. For cities in the central and western regions, the key to improving the IGDE is to extend and renew existing industries to achieve clean, high-end transformation and upgrading. At the same time, blindly undertaking the transfer of eastern industries for economic growth without considering environmental benefits should be avoided. For eastern cities, where the economic development momentum is strong, it is necessary to find a balance between the traditional economic growth momentum and the new model, the establishment of a systematic and complete institutional system should be accelerated to promote the IGDE, and healthy and sustainable economic development should be achieved. In addition to paying attention to the differences between the east and the west, the differences between the north and the south should also be taken seriously.

A green technology innovation system should be built, and green production in industries should be promoted. The level of scientific and technological innovation has a negative effect on IGDE. Therefore, accelerating the construction of green technology innovation system is conducive to its development. First of all, the main body of green technology innovation should be fostered and strengthened. Support for technological innovation should be strengthened, and green technology innovation alliances should be built, led by enterprises, in collaboration with colleges, research institutes, and financial capital to promote the deep integration of innovation entities. Second, the transformation of innovation achievements in green technology should be accelerated. Enterprises, colleges, and research institutions should be supported in establishing green technology innovation project incubation, whereas green investment and local venture capital funds should be guided to support the transformation and application of achievements. In addition, a distinctive regional and specialized green technology trading market should be established, and the transformation of green technology innovation achievements should be promoted through market means. Lastly, the environment should be optimized for green technology innovation. By improving the intellectual property protection system for green technologies and ensuring intellectual property protection in all aspects from research and development (R&D) to the application of green technologies, and by guiding financial institutions to support green technology innovation enterprises and project financing, an enabling environment can be created for innovation. The industrial structure also has a negative inhibitory effect. It is necessary to establish a sound ecological economic system with ecological industrialization as the main body. On the one hand, ecological industrialization requires promoting the ecological transformation of traditional industries. In line with the requirements of green, low-carbon and circular development, traditional industries such as petrochemical, building materials, and nonferrous metals should be actively transformed and upgraded. We can increase the reform, innovation, and development of new drivers, as well as cut back on shutting down outdated production facilities to promote green development. On the other hand, ecological industrialization requires that ecological and environmental protection should also be industrialized. We should develop environmental protection industries, clean energy industries, and clean production industries, improve economic efficiency and ecological benefits, vigorously develop the circular economy, and cultivate new economic growth points. In particular, the industrial structure of cities south of the Yangtze River Economic Belt has a significant impact on the IGDE. Therefore, we should combine the resource endowments, industrial foundations, and geographical advantages of different regions to formulate corresponding industrial green development strategies to promote the transformation and upgrading of industrial structure. For the Pearl River Delta region, we should give full play to the agglomeration effect of advanced production factors and actively apply advanced technologies such as the internet, big data, and artificial intelligence to transform and upgrade traditional manufacturing industries, as well as promote industrial transformation and upgrading. At the same time, the development of new models of new manufacturing and service-oriented manufacturing should be accelerated, new energy, new materials, data information, energy conservation, and environmental protection should be actively cultivated, in addition to other strategic emerging industries, and industrial transformation and structural adjustment and optimization should be promoted in the pan-Pearl River Delta as a leader.

In combination with the development trend of population scale, differentiated urban development strategies should be implemented. In the current process of urbanization, some cities have problems of population loss and economic growth slowing down. It is clear that urban expansion and contraction are both inevitable stages of urban development, and differentiation strategies should be implemented for cities and towns that are expanding, stable, and contracting. For large cities with expanding population, such as the Yangtze River Delta and Pearl River Delta, according to the carrying capacity of urban environment, we should scientifically delimit the urban growth boundary, optimize the urban spatial structure, combine with the local development foundation, introduce efficient environmental protection industries, and promote the formation of a coordinated spatial pattern of production, living, and ecology. For urban population scales that are relatively stable, such as the central region, because the population and urban land will not surge in the short term, attention should be paid to the green transformation of existing industries to improve the level of green industry. Meanwhile, the public service system should be established and improved, the capacity to provide public services should be improved, and we should focus on improving the quality of the city to avoid losing people. For cities with shrinking populations, such as northeast China and western China, a series of problems such as population loss, idling of land and facilities, and industrial decline caused by the transformation of industrial bases, decline of old cities, and hollowing out of rural areas in some regions should be correctly considered. The low-quality, low-efficiency, inadequate utilization and unreasonable stock of urban construction land, such as idle industrial land that does not meet the requirements of production safety and environmental protection, should be renovated, improved, rebuilt, activated, and upgraded to create new space for urban industrial development and urban construction. In addition, public infrastructure commensurate with the size of the population should be built to guide the concentration of population, industry, and other factors within the city.

## Figures and Tables

**Table 1:** Input–output index system of industrial green development efficiency (IGDE).

Target Layer	Rule Layer	Factor Layer	Index Layer
Input–output index system of IGDE	Input	Labor input	Quantity of employment
		Capital input	Fixed investments
	Desirable Output	Industrial output value	The added value of three industries
	Undesirable Output	Wastewater	The discharge of industrial waste water
		Waste gas	The discharge of industrial SO_2_
		Waste residue	The discharge of industrial smoke and dust

**Table 2:** IGDE values of China’s four regions from 2008 to 2018.

IGDE Values	2008	2009	2010	2011	2012	2013	2014	2015	2016	2017	2018
National	0.5346	0.4478	0.4125	0.4728	0.4695	0.5033	0.4335	0.3634	0.3633	0.3682	0.3925
Eastern	0.6044	0.5369	0.4671	0.5217	0.5127	0.5850	0.4814	0.4032	0.3773	0.3806	0.4083
Central	0.4637	0.3767	0.3567	0.3946	0.3973	0.4095	0.3550	0.2666	0.2679	0.2840	0.2995
Western	0.5545	0.4602	0.4410	0.5043	0.5109	0.5161	0.4450	0.3856	0.3746	0.3773	0.3959
Northeast	0.4981	0.3792	0.3554	0.4825	0.4550	0.5129	0.4926	0.4670	0.5546	0.5387	0.5904

**Table 3:** Comparison of OLS, GWR, and multiscale geographical weighted regression (MGWR) model indicators.

Model Indices	MGWR	GWR	OLS
*R* ^2^	0.617	0.579	0.426
AICc	641.407	647.581	680.373
Residual Sum of Squares (RSS)	115.910	116.459	165.809
Number of effective parameters	36.035	44.052	/

**Table 4:** GWR and MGWR bandwidth comparison.

Variable	The Bandwidth of MGWR	The Bandwidth of GWR
Constant term	118	140
Economic development.	92	140
Consumption level	287	140
Technological innovation	288	140
Industrial structure	131	140
Population density	82	140
Urbanization level	167	140
Government regulation	276	140
Openness	288	140

**Table 5:** Statistical description of MGWR regression coefficient.

Variable	Min	Median	Max	Mean	Standard Deviation
Constant term	−0.286	−0.036	0.399	−0.021	0.158
Economic development	0.253	0.600	0.855	0.549	0.162
Consumption level	0.195	0.219	0.254	0.219	0.013
Technological innovation	−0.150	−0.135	−0.120	−0.135	0.004
Industrial structure	−0.476	−0.056	0.103	−0.107	0.150
Population density	−0.307	0.148	0.469	0.141	0.142
Urbanization level	−0.243	−0.131	0.028	−0.117	0.072
Government regulation	0.268	0.282	0.408	0.295	0.034
Openness	−0.061	−0.053	−0.048	−0.053	0.003

## Data Availability

The data presented in this study are openly available from the National Bureau of Statistics, reference numbers 978-7-5037-9120-8, 978-7-5037-8770-6, 978-7-5037-8432-3, 978-7-5037-8082-0, 978-7-5037-7706-6, 978-7-5037-7350-1, 978-7-5037-7019-7, and 978-7-5037-6754-8.

## References

[1] Lloyd E.A., Shepherd T.G. (2020). Environmental catastrophes, climate change, and attribution. Ann. N. Y. Acad. Sci..

[2] Song Z.G. (2021). Economic growth and carbon emissions: Estimation of a panel threshold model for the transition process in China. J. Clean. Prod..

[3] Ministry of Industry and Information Technology of the People’s Republic of China Green Development Plan for Industry (2016–2020). https://www.miit.gov.cn/jgsj/jns/gzdt/art/2020/art_4290757b7785460795cc49f4fc3ecba4.html.

[4] National Bureau of Statistics Statistical Bulletin of the People’s Republic of China on National Economic and Social Development 2019. http://www.yunfu.gov.cn/yftjj/gkmlpt/content/1/1299/post_1299760.html.

[5] Pearce D.W., Barbiere M.A. (1989). Blueprint for A Green Economy.

[6] Kates R.W., Parris T.M., Leiserowitz A.A. (2005). What is sustainable development? Goals, indicators, values, and practice. Environment.

[7] Chen Y., Chen C.Y., Hsieh T. (2011). Exploration of sustainable development by applying green economy indicators. Environ. Monit. Assess..

[8] World Bank (2012). Inclusive Green Growth: The Pathway to Sustainable Development.

[9] Cui H.R., Liu Z.L. (2021). Spatial-Temporal Pattern and Influencing Factors of the Urban Green Development Efficiency in Jing-Jin-Ji Region of China. Pol. J. Environ. Stud..

[10] Chen C., Wang K., Feng M. (2020). Research on China’s Industrial Green Development based on the Pressure-State-Response Model. J. Sci. Ind. Res..

[11] Farrell M.J. (1957). The Measurement of Productive Efficiency. J. R. Statist. Soc..

[12] Li P., Tong L.J., Guo Y.H., Guo F.Y. (2020). Spatial-temporal Characteristics of Green Development Efficiency and Influencing Factors in Restricted Development Zones: A Case Study of Jilin Province, China. Chin. Geogr. Sci..

[13] Ramakrishnan R. (2005). An analysis of energy consumption and carbon dioxide emissions in countries of the Middle East and North Africa. Energy.

[14] Zhou P., Ang B.W., Han J.Y. (2009). Total factor carbon emission performance: A Malmquist index analysis. Energy Econ..

[15] Liu Y., Yang J.L., Liang Y. (2019). Efficiency Evaluation and Equilibrium Characteristics of Green Development in Chinese urban agglomerations. Econ. Geogr..

[16] Liu Z., Zhang H., Zhang Y.J. (2020). How does industrial policy affect the eco-efficiency of industrial sector? Evidence from China. Apply Energy.

[17] Ezici B., Egilmez G., Gedik R. (2020). Assessing the eco-efciency of U.S. manufacturing industries with a focus on renewable vs. nonrenewable energy use: An integrated time series MRIO and DEA approach. J. Clean. Prod..

[18] Park J., Park J.M., Park H.S. (2019). Scaling-Up of Industrial Symbiosis in the Korean National Eco-Industrial Park Program: Examining Its Evolution over the 10 Years between 2005–2014. J. Ind. Ecol..

[19] Haider S., Danish M.S., Sharma R. (2019). Assessing energy efciency of Indian paper industry and infuencing factors: A slack-based firm-level analysis. Energy Econ..

[20] Tenente M., Henriques C., Silva P.P. (2020). Eco-efciency assessment of the electricity sector: Evidence from 28 European Union countries. Econ. Anal. Policy.

[21] Aigner D.J., Lovell C.A.K., Schmidt P. (1977). Formulation and estimation of stochastic frontier production function models. J. Econom..

[22] Battese G.E., Coelli T.J. (1988). Prediction of farm-level technical efficiencies with a generalized frontier production function and panel data. J. Econom..

[23] Chen W., Ning S.Y., Chen W.J., Liu E.N., Wang Y.N., Zhao M.J. (2020). Spatial-temporal characteristics of industrial land green efficiency in China: Evidence from prefecture-level cities. Ecol. Indic..

[24] Liu R.Y., Wang D.Q., Zhang L., Zhang L.H. (2019). Can green financial development promote regional ecological efficiency? A case study of China. Nat. Hazards.

[25] Gao Y., Tsai S.B., Xue X.Q., Ren T.Z., Du X.M., Chen Q., Wang J.T. (2018). An Empirical Study on Green Innovation Efficiency in the Green Institutional Environment. Sustainability.

[26] Fu J.P., Xiao G.R., Guo L.L., Wu C.Y. (2018). Measuring the Dynamic Efficiency of Regional Industrial Green Transformation in China. Sustainability.

[27] Guo Y.H., Tong L.J., Mei L. (2020). The effect of industrial agglomeration on green development efficiency in Northeast China since the revitalization. J. Clean. Prod..

[28] Zhu B.Z., Zhang M.F., Zhou Y.H., Wang P., Sheng J.C., He K.J., Wei Y.M., Xie R. (2019). Exploring the effect of industrial structure adjustment on interprovincial green development efficiency in China: A novel integrated approach. Energy Policy.

[29] Wang X.Q., Wu Q.M., Majeed S., Sun D.H. (2018). Fujian’s Industrial Eco-Efficiency: Evaluation Based on SBM and the Empirical Analysis of lnfluencing Factors. Sustainability.

[30] Lu Y.Y., Cao B., Hua Y.D., Ding L. (2020). Efficiency Measurement of Green Regional Development and Its Influencing Factors: An Improved Data Envelopment Analysis Framework. Sustainability.

[31] Sun Z.Q., Sun T. (2019). Financial Development, Industrial Structure Optimization, and Eco-efficiency Promotion. Fresenius Environ. Bull..

[32] Tone K.A. (2002). slacks-based measure of super-efficiency in data envelopment analysis. Eur. J. Oper. Res..

[33] Liu K., Qiao Y.R., Shi T., Zhou Q. (2020). Study on coupling coordination and spatiotemporal heterogeneity between economic development and ecological environment of cities along the Yellow River Basin. Environ. Sci. Pollut. Res..

[34] Shen T.Y., Yu H.C., Zhou L., Gu H.Y., He H.H. (2020). Influence Mechanism of Second-hand Housing Price in Beijing—A Study based on Multi-scale Geo-weighted Regression Model (MGWR). Econ. Geogr..

[35] Huang L., Wu C.Q. (2019). Study on urban industrial green development efficiency and its spatial driving mechanism in the Yangtze River Economic Belt. China Popul. Resour. Environ..

[36] Wang K.L., Huang Q.Q., Meng X.R. (2017). Industrial ecological efficiency of mining cities based on environmental pressure. Syst. Eng..

[37] Ott I., Soretz S. (2018). Green Attitude and Economic Growth. Environ. Resour. Econ..

[38] Wang G., Liu S. (2020). Is technological innovation the effective way to achieve the “double dividend” of environmental protection and industrial upgrading?. Environ. Sci. Pollut. Res..

[39] Xie H. (2020). Spatial Heterogeneity Strategies for Pollution Agglomeration Control in China: Based on the Coordination Between Industrialization and Urbanization. Arab. J. Geosci..

[40] Eriksson C., Zehaie F. (2005). Population Density, Pollution and Growth. Environ. Resource. Econ..

[41] Kolomak E.A. (2012). Assessment of the urbanization impact on economic growth in Russia. Reg. Res. Russ..

[42] Liu C., Ma C., Xie R. (2020). Structural, Innovation and Efficiency Effects of Environmental Regulation: Evidence from China’s Carbon Emissions Trading Pilot. Environ. Resource. Econ..

[43] Wang Z.Y., Zhang M.Y., Wang Y.X., Fan Y.X. (2019). Spatial and temporal Differentiation of China’s Marine economic elasticity and analysis of influencing Factors. Econ. Geogr..

[44] Lee J. (2013). The contribution of foreign direct investment to clean energy use, carbon emissions and economic growth. Energy Policy.

[45] Wang X., Li Y. (2020). Research on measurement and improvement path of industrial green development in China: A perspective of environmental welfare efficiency. Environ. Sci. Pollut. Res..

[46] Qiu F., Chen Y., Tan J., Liu J., Zheng Z., Zhang X. (2020). Spatial-temporal Heterogeneity of Green Development Efficiency and Its Influencing Factors in Growing Metropolitan Area: A Case Study for the Xuzhou Metropolitan Area. Chin. Geogr. Sci..

[47] Chen Y., Zhu B., Sun X., Xu G. (2020). Industrial environmental efficiency and its influencing factors in China: Analysis based on the Super-SBM model and spatial panel data. Environ. Sci. Pollut. Res..

[48] Alam M.S., Atif M., Chien-Chi C., Soytaş U. (2019). Does corporate R&D investment affect firm environmental performance? Evidence from G-6 countries. Energy Econ..

[49] Shen Y., Sun S., Yue S., Sun X. (2020). Ecological development efficiency index of tropics and subtropics in China. Environ. Sci. Pollut. Res..

[50] Wang J., Zhao T., Zhang X. (2016). Environmental assessment and investment strategies of provincial industrial sector in China: Analysis based on DEA model. Environ. Impact Asses..

[51] Yang G., Zhang F., Zhang F., Ma D., Gao L., Chen Y., Luo Y., Yang Q. (2021). Spatiotemporal changes in efficiency and influencing factors of China’s industrial carbon emissions. Environ. Sci. Pollut. Res..

